# Peripheral blood biomarkers predict immune-related adverse events in non-small cell lung cancer patients treated with pembrolizumab: a multicenter retrospective study

**DOI:** 10.7150/jca.53242

**Published:** 2021-02-16

**Authors:** Saeka Egami, Hitoshi Kawazoe, Hironobu Hashimoto, Ryuji Uozumi, Toko Arami, Naomi Sakiyama, Yuichiro Ohe, Hideo Nakada, Tohru Aomori, Shinnosuke Ikemura, Koichi Fukunaga, Masakazu Yamaguchi, Tomonori Nakamura

**Affiliations:** 1 Division of Pharmaceutical Care Sciences, Center for Social Pharmacy and Pharmaceutical Care Sciences, Keio University Faculty of Pharmacy, 1-5-30 Shibakoen, Minato-ku, Tokyo 105-8512, Japan.; 2 Division of Pharmaceutical Care Sciences, Keio University Graduate School of Pharmaceutical Sciences, 1-5-30 Shibakoen, Minato-ku, Tokyo 105-8512, Japan.; 3 Department of Pharmacy, National Cancer Center Hospital, 5-1-1 Tsukiji, Chuo-ku, Tokyo 104-0045, Japan.; 4 Department of Biomedical Statistics and Bioinformatics, Kyoto University Graduate School of Medicine, 54 Kawahara-cho, Shogoin, Sakyo-ku, Kyoto 606-8507, Japan.; 5 Department of Thoracic Oncology, National Cancer Center Hospital, 5-1-1 Tsukiji, Chuo-ku, Tokyo 104-0045, Japan.; 6 Division of Hospital Pharmacy Science, Keio University Faculty of Pharmacy, 1-5-30 Shibakoen, Minato-ku, Tokyo 105-8512, Japan.; 7 Department of Pharmacy, Keio University Hospital, 35 Shinanomachi, Shinjuku-ku, Tokyo 160-8582, Japan.; 8 Keio Cancer Center, Keio University School of Medicine, 35 Shinanomachi, Shinjuku-ku, Tokyo 160-8582, Japan.; 9 Division of Pulmonary Medicine, Department of Medicine, Keio University School of Medicine, 35 Shinanomachi, Shinjuku-ku, Tokyo 160-8582, Japan.

**Keywords:** immune checkpoint inhibitor, blood cell count, absolute lymphocyte count, neutrophil-to-lymphocyte ratio, lymphocyte-to-monocyte ratio, platelet-to-lymphocyte ratio.

## Abstract

**Background:** Pembrolizumab is currently the standard treatment for patients with advanced non-small cell lung cancer (NSCLC). However, the association between immune-related adverse events (irAEs) and peripheral blood cell counts remains unclear. We aimed at identifying peripheral blood cell counts that may predict the development of pembrolizumab-induced irAEs.

**Methods:** We retrospectively analyzed data on consecutive patients with advanced NSCLC who received pembrolizumab monotherapy as first-line or later-line therapy at the National Cancer Center Hospital and Keio University Hospital. We used data between December 2015 and November 2018. The primary endpoint was the relationship between peripheral blood cell count data and early-onset irAEs during the 6-weeks study period. Receiver operating characteristic (ROC) curve and multivariable logistic regression analyses were performed.

**Results:** In total, 92 patients were evaluated, of whom 45 (48.9%) had at least one irAE during the first 6-weeks after treatment initiation. The ROC curves revealed that the optimal cutoff of pretreatment absolute lymphocyte count (ALC), neutrophil-to-lymphocyte ratio (NLR), lymphocyte-to-monocyte ratio (LMR), and platelet-to-lymphocyte ratio (PLR) for onset of irAEs were 1459, 2.320, 1.538, and 165, respectively. Multivariable logistic regression analyses revealed that pretreatment ALC>1450 and LMR>1.6 were significantly associated with a reduced risk for onset of any irAEs, whereas pretreatment NLR>2.3 and PLR>165 were significantly associated with an increased risk.

**Conclusions:** The findings suggest that considering the routine availability of blood cell count data before the initiation of treatment with pembrolizumab, it may be useful in identifying early-onset irAEs during the 6-weeks study period in clinical practice.

## Introduction

Lung cancer is the most commonly occurring malignancy worldwide, and is the leading cause of cancer-related deaths 1. Several pivotal phase III trials have shown that immune-checkpoint inhibitor (ICI) therapy is the current standard treatment for patients with advanced non-small cell lung cancer (NSCLC) 2-5. However, ICIs induce immune-related adverse events (irAEs), the development of which remains an unresolved issue in clinical practice 6, 7. The incidence rate of irAE ranges from 16.0% to 68.2% in such settings 8-22. Moreover, there is currently a lack of sufficient data on predictive biomarkers for irAEs in patients receiving ICIs in clinical practice 8, 9.

According to previous retrospective studies, irAEs induced by nivolumab or pembrolizumab—both monoclonal antibodies that target programmed cell death protein 1 (PD-1)—are associated with survival benefit in patients with advanced NSCLC and malignant melanoma 10-18. Circulating blood inflammatory biomarkers, such as absolute lymphocyte count (ALC), neutrophil-to-lymphocyte ratio (NLR), lymphocyte-to-monocyte ratio (LMR), and platelet-to-lymphocyte ratio (PLR) have the potential to predict treatment efficacy in advanced NSCLC 19-22. Taken together, we hypothesized that peripheral blood cell counts can predict pembrolizumab-induced irAEs in patients with advanced NSCLC. Pembrolizumab-induced irAEs, in addition to having a negative impact on patients' quality of life (QOL), also pose a significant burden in terms of healthcare resources and costs. The early detection of irAEs is essential for clinicians to minimize the burden on patients' QOL. However, the relationship of peripheral blood cell counts at baseline and at 3 weeks after treatment initiation with early-onset irAEs as well as clinical treatment efficacy has not yet been investigated in patients with advanced NSCLC treated with pembrolizumab monotherapy in a large-scale, multicenter study.

The present study, therefore, aimed to evaluate whether routinely available blood cell counts could have a predictive value in the development of pembrolizumab-induced irAEs.

## Methods

### Patients

This multicenter, retrospective, observational study was conducted at the National Cancer Center Hospital, a high-volume cancer center in Tokyo; Keio University Hospital, a tertiary hospital in Tokyo; and Keio University Faculty of Pharmacy, a private pharmacy school in Tokyo. Research members from Keio University Faculty of Pharmacy retrieved data from the electronic medical records at the National Cancer Center Hospital and Keio University Hospital. Data integration was then performed at Keio University Faculty of Pharmacy, and subsequent statistical analyses were performed at Kyoto University Graduate School of Medicine. The multicenter observational methodology of this study was the same as that followed in a previous study conducted by our coauthors 23. The inclusion criteria were as follows: 1) consecutive patients aged ≥ 20 years with postoperative recurrence or unresectable stage III and IV NSCLC, 2) patients with disease control and alive at 6-weeks after pembrolizumab monotherapy (200 mg/body every 3 weeks) administered as a first-line or later-line treatment between December 2015 and November 2018, and 3) patients without complications or prior history of chronic or recurrent autoimmune disease and interstitial pulmonary disease. The treatment schedule and follow-up were modified at the clinicians' discretion according to patients' toxicity profiles. Clinic visits and imaging evaluations were also generally conducted from 6 to 8 weeks according to the RECIST criteria, version 1.1. Patient records were de-identified and analyzed anonymously.

The collected data included patients' baseline age, sex, Eastern Cooperative Oncology Group performance status, treatment line, programmed cell death ligand 1 (PD-L1) expression, and routinely available blood cell count data, including ALC, absolute neutrophil counts, absolute monocyte counts, and platelet counts at baseline (defined as the most recent blood count within 1 week before the initiation of treatment) and at 3 weeks after the start of treatment, as well as incidence and types of irAEs. The tumor PD-L1 expression level was examined in archived biopsy samples using the PD-L1 IHC 22C3 pharmDx (Dako) kit (Agilent Technology, Inc., Santa Clara, CA, USA) according to the manufacturer's protocol. In the present study, we adopted irAEs, as routinely assessed by physicians. Any irAEs that occurred 6-weeks after pembrolizumab initiation were not counted. In addition, infusion reactions, which can be observed with the use of any monoclonal antibody, were not included as irAEs according to previous studies 12, 24, 25.

Patients were excluded from the study based on the following criteria: 1) history of prior administration of any ICIs and/or investigational drugs as part of a clinical trial or at a previous hospital before the investigation period, 2) discontinuation of treatment because of death or hospital transfer before 6 weeks, 3) discontinuation of treatment after the first cycle because of disease progression or adverse events, 4) lack of laboratory data at 3 weeks after the first cycle (acceptable range from day 19 to 23), and 5) study participation shorter than 6 weeks (i.e., patients that started treatment between October 2018 and November 2018).

The study protocol was approved by the ethics committees of the National Cancer Center Hospital (approval number: 2019-199), Keio University Hospital (approval number: 20180313), and Keio University Faculty of Pharmacy (approval number: 200918-2), and was conducted in accordance with the Declaration of Helsinki and the Ethical Guidelines for Medical and Health Research involving Human Subjects by the Ministry of Education, Culture, Sports, Science, and Technology and the Ministry of Health, Labour, and Welfare of Japan. The Japanese law does not require the obtainment of individual informed consent from participants in non-invasive observational trials, such as the present study. Therefore, we used the National Cancer Center Hospital and Keio University Hospital official website as an opt-out method rather than acquiring written or oral informed consent.

### Endpoints

The primary endpoint was the association between early onset irAEs and the peripheral blood cell counts (both at baseline and at 3 weeks after pembrolizumab therapy). Similar to potential peripheral blood biomarkers reported in several previous studies 19-22, data on blood cell counts were used for the calculation of the ALC, NLR, LMR, and PLR. The changes in ALC, NLR, LMR, and PLR were evaluated by comparing the posttreatment values with their respective pretreatment values.

### Statistical analysis

The receiver operating characteristic (ROC) curve analyses were used for the identification of the cutoff values of the above-mentioned potential peripheral blood cell count indices for early-onset irAEs. The optimal cut-off values were determined using Youden's index 26. Youden's index was calculated as the maximum value using the following formula: sensitivity - (1 - specificity). Subsequently, multivariable logistic regression analyses were used to assess the cutoff peripheral blood cell count values associated with early-onset irAEs. Potential explanatory variables pertaining to the patients' backgrounds, such as age and PD-L1 expression (≥50%) were included in the multivariable models. According to a previous report, Shah et al. 27 reported a paradoxical, but not direct, correlation between patients' age and incidence of irAEs. In addition, PD-L1 expression level was a predictive biomarker only for the efficacy of pembrolizumab. These explanatory variables were determined by clinical judgment of our coauthors.

All statistical analyses were performed using SAS version 9.4 and JMP 15.0.0 (SAS Institute, Inc., Cary, NC, USA). All *p* values were two-sided, and *p* < 0.05 was considered significant.

## Results

### Patient characteristics

**Figure 1:**
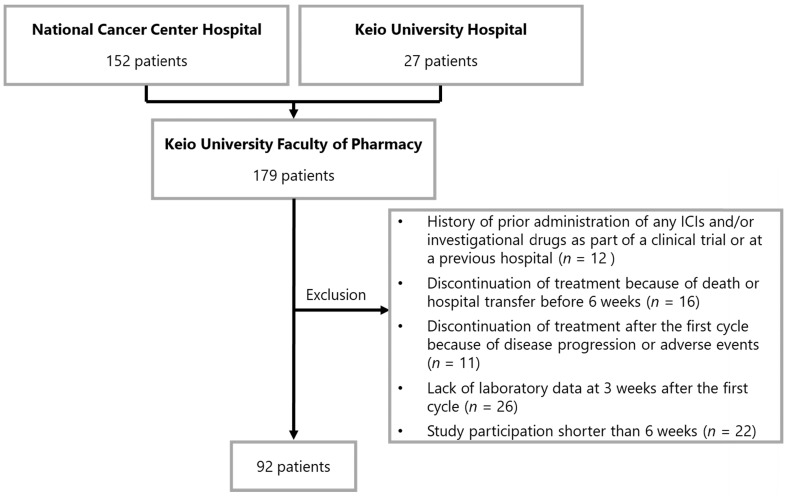
** Patient enrollment flowchart.** Abbreviations: ICI, immune checkpoint inhibitor.

A flowchart illustrating the patient enrollment process is shown in Figure 1. None of the patients were lost during follow-up. On the basis of the exclusion criteria, 87 patients were withdrawn from the analysis. Data of 92 patients were evaluated in this study. The baseline patient characteristics are summarized in Table 1. The median age of the patients was 60 years (interquartile range [IQR]: 34-85 years). The number of patients who received 1st line, 2nd line, and ≥ 3rd line treatment was 62 (67.4%), 22 (23.9%), and eight (8.7%), respectively. In terms of PD-L1 expression level, the numbers of patients with values ≥50%, 1-49%, and <1% were 74 (80.4%), 17 (18.5%), and 1 (1.1%), respectively.

### Endpoints

A total of 45 patients (48.9%) showed early irAE onset, of whom 35 (38.0%) had skin reactions. All irAEs are listed in Table 2. The median value of pretreatment lymphocyte count, neutrophil count, monocyte count, and platelet count was 1464 cells/mm^3^ (IQR: 1112-1819 cells/mm^3^), 5200 cells/mm^3^ (IQR: 3606-6680 cells/mm^3^), 470 cells/mm^3^ (IQR: 350-634 cells/mm^3^), and 29×10^4^ cells/mm^3^ (IQR: 22×10^4^-36×10^4^ cells/mm^3^), respectively. The median value of post treatment lymphocyte count, neutrophil count, monocyte count, and platelet count was 1447 cells/mm^3^ (IQR: 1110-1898 cells/mm^3^), 4625 cells/mm^3^ (IQR: 3546-5876 cells/mm^3^), 467 cells/mm^3^ (IQR: 369-574 cells/mm^3^), and 30×10^4^ cells/mm^3^ (IQR: 25×10^4^-39×10^4^ cells/mm^3^), respectively.

**Figure 2:**
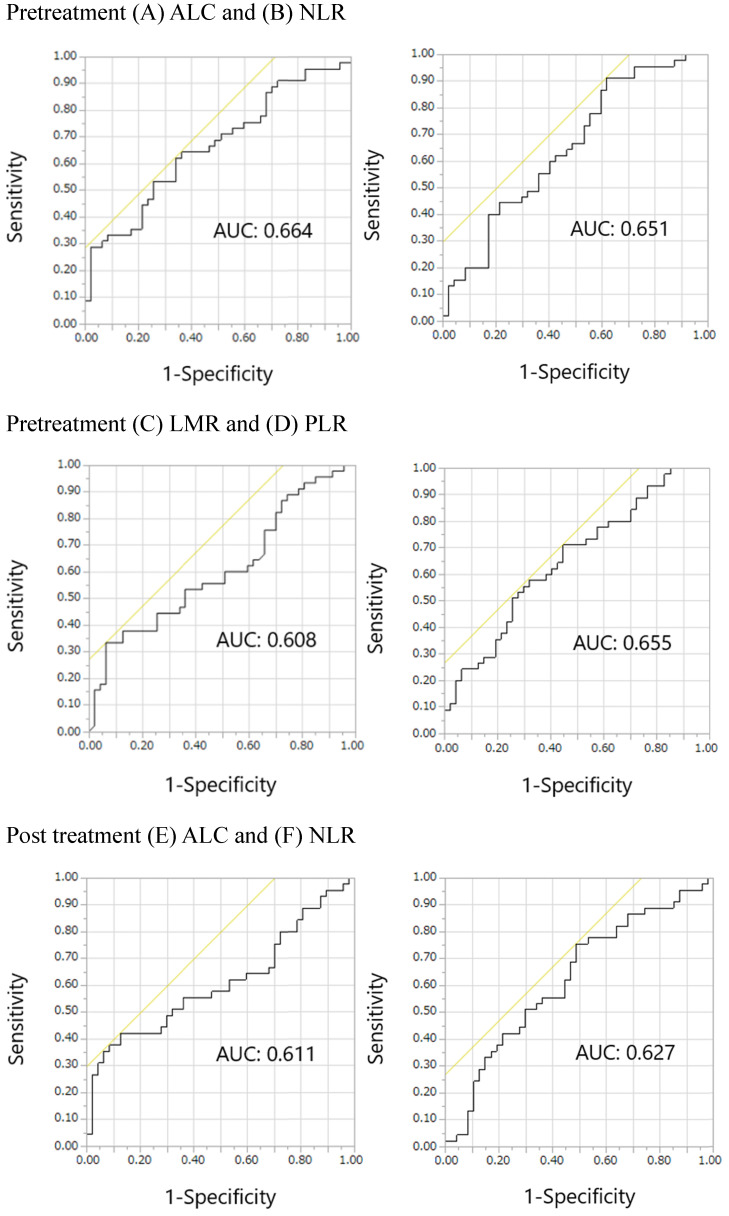

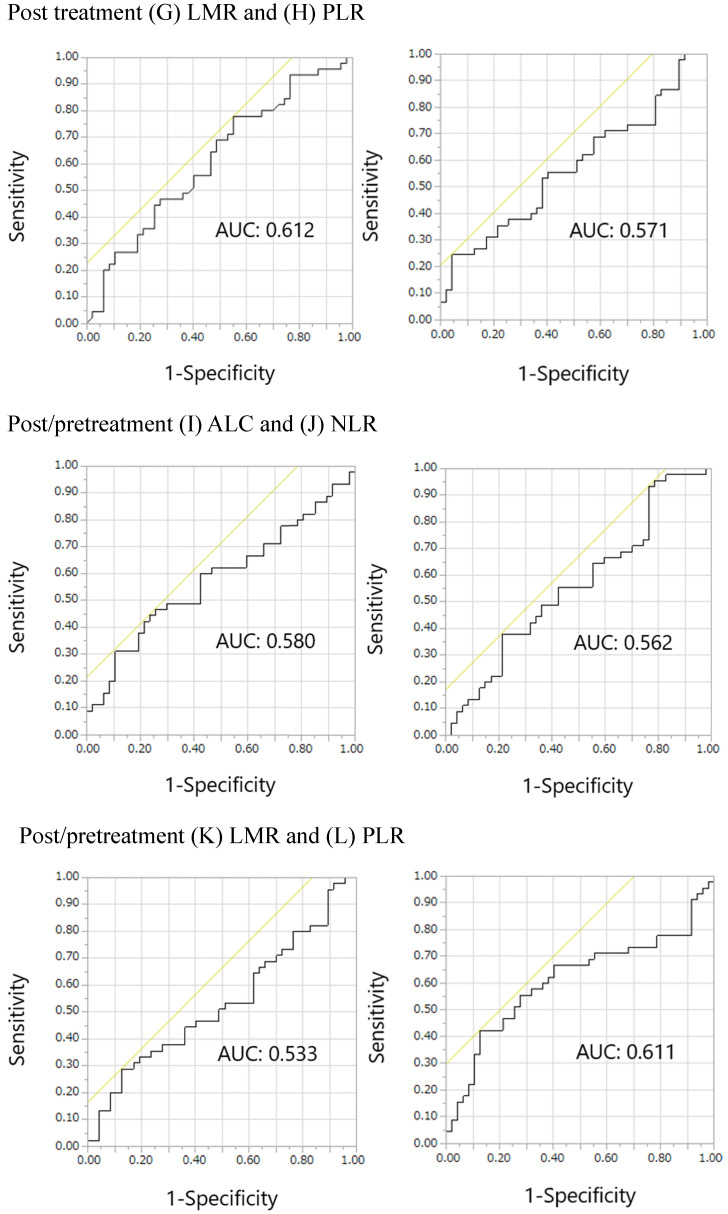
** Receiver operating characteristic curves for the early onset of any immune-related adverse events.** Pretreatment (A) ALC, (B) NLR, (C) LMR, and (D) PLR; Posttreatment (E) ALC, (F) NLR, (G) LMR, and (H) PLR; Post/pretreatment (I) ALC, (J) NLR, (K) LMR, and (L) PLR. Abbreviations: ALC, absolute lymphocyte count; NLR, neutrophil-to-lymphocyte ratio; LMR, lymphocyte-to-monocyte ratio; PLR, platelet-to-lymphocyte ratio; AUC, area under the curve.

The ROC curves for pretreatment ALC, NLR, LMR, and PLR, post treatment ALC, NLR, LMR, and PLR, and post/pretreatment ALC, NLR, LMR, and PLR are shown in Figure 2. The areas under the curve (AUCs) of pretreatment ALC, NLR, LMR, and PLR were 0.664, 0.651, 0.608, and 0.655, respectively. The AUCs of post treatment ALC, NLR, LMR, and PLR were 0.611, 0.627, 0.612, and 0.571, respectively. The AUCs of post/pretreatment ALC, NLR, LMR, and PLR were 0.580, 0.562, 0.533, and 0.611, respectively. Overall, pretreatment ALC, NLR, LMR, and PLR showed relatively large AUCs in the ROC curves of irAE onset compared with others. Thus, we focused on pretreatment, but not posttreatment and post/pretreatment values, prognostically. The optimal cutoff pretreatment ALC, NLR, LMR, and PLR values were 1459, 2.320, 1.538, and 165, respectively, while the corresponding Youden's index values were 0.283, 0.294, 0.270, and 0.264, respectively. Similarly, pretreatment ALC, NLR, LMR, and PLR showed relatively large AUCs in the ROC curves of skin reactions compared with others (Supplemental online Figure 1). The AUCs of pretreatment ALC, NLR, LMR, and PLR were 0.637, 0.593, 0.551, and 0.660, respectively. The optimal cutoff pretreatment ALC, NLR, LMR, and PLR values were 1336, 2.320, 1.500, and 204, respectively. The Youden's index values of pretreatment ALC, NLR, LMR, and PLR were 0.245, 0.248, 0.163, and 0.295, respectively.

As shown in Table 3, multivariable logistic regression analyses revealed that pretreatment ALC>1450 and LMR>1.6 were significantly associated with a reduced risk of onset of any irAEs (adjusted odds ratio [OR]: 0.24, 95% confidence interval [CI]: 0.09-0.61, *p* = 0.003; adjusted OR: 0.12, 95% CI: 0.03-0.52, *p* = 0.004, respectively), whereas pretreatment NLR>2.3 and PLR>165 was significantly associated with an increased risk of onset of any irAEs (adjusted OR: 5.99, 95% CI: 1.73-20.74, *p* = 0.005; adjusted OR: 2.87, 95% CI: 1.16-7.08, *p* = 0.022). The multivariable logistic model estimated the AUCs of pretreatment ALC, NLR, LMR, and PLR>165 at 0.752, 0.718, 0.735, and 0.706, respectively.

## Discussion

In the present study, we found that pretreatment ALC>1450 and LMR>1.6 were significantly associated with a reduced risk of onset of irAEs, whereas pretreatment NLR>2.3 and PLR>165 were significantly associated with an increased risk in patients with advanced NSCLC. Currently, there is a lack of sufficient data on biomarkers that may be predictive of irAEs in patients receiving ICIs. We hypothesized that peripheral blood cell counts could predict pembrolizumab-induced irAEs in patients with advanced NSCLC; and, with the present study's findings, our hypothesis was met.

To the best of our knowledge, this is the first study to investigate whether routinely available blood cell counts could predict pembrolizumab-induced irAEs. The following studies have investigated patients treated with nivolumab, mainly because it is the first class of ICIs to be administered. While clinical researchers have focused on the association between several peripheral blood biomarkers and the treatment efficacy of ICIs, irAEs have not been investigated extensively in this context 19-22. Diehl et al. 8 first reported that in patients with solid tumors treated with nivolumab (*n* = 125) or pembrolizumab (*n* = 42), with or without concurrent ipilimumab, an ALC>2000 at baseline and an ALC>2000 at one month after treatment initiation were significantly associated with the development of grade 2 or higher irAEs. Recently, Pavan et al. 9 also reported that in patients with advanced NSCLC who received nivolumab (*n* = 145), pembrolizumab (*n* = 32), and atezolizumab (*n* = 7), a baseline NLR<3 and PLR<180 was significantly associated with the development of irAEs. Our data showed that pretreatment higher ALC values were significantly associated with a reduced risk of irAEs, whereas higher NLR and PLR values were significantly associated with an increased risk of irAEs. However, the exact reason for this discrepancy with the previous published experiences remains unclear 8, 9. First, there was difference in the ICI treatment line; our data was mainly based on the 1st treatment (67.4%). In contrast, Pavan et al. 9 study comprised mainly ≥ 2nd line treatment (85.9%). Thus, there may be bone marrow exhaustion due to prior treatment with cytotoxic chemotherapy. Second, there was difference in the patients' ethnicity—Japanese and Western. Overall, the incidence rate of irAEs in Japan is relatively higher than that in other countries 8-22. Third, previous studies mainly investigated patients treated with nivolumab as opposed to our study.

To account for the presence of lead-time bias associated with the time-dependent development of irAEs, we selected a 6-weeks study period in accordance with that in previous studies 10, 11. Another reason why the 6-week period was chosen was the occurrence rate of irAEs and the timing of computerized tomography. The rates of irAEs are around 50% until 6 weeks after the initiation of ICI therapy 10-14, 21. The follow-up period for advanced NSCLC patients in terms of imaging was generally from 6 week to 8 week in Japan 10. Additionally, an earlier report by our coauthors focused on the association between post/pretreatment LMR and NLR values (2 and 4 weeks after nivolumab treatment) and the treatment efficacy of nivolumab as well as early irAEs (defined as the presentation of these events within 4 weeks of the start of nivolumab treatment) 21. However, the association of those peripheral blood biomarkers at baseline and 3 weeks after the initiation of pembrolizumab with early-onset irAEs has not yet been investigated in patients with advanced NSCLC treated with pembrolizumab monotherapy in a multicenter study. This study suggests that pretreatment ALC, NLR, LMR, and PLR values may predict the early onset of any irAEs in patients with advanced NSCLC. There was a discrepancy between ALC and LMR results, and NLR and PLR results. We believe that lymphocyte count is a key parameter; ALC and LMR have lymphocyte count as a numerator, whereas NLR and PLR have lymphocyte count as a denominator. Interestingly, whereas the baseline results of our multicenter study were in line with those of previous studies 8, 9, our posttreatment peripheral blood cell count values were not. These findings suggest that considering pretreatment peripheral blood cell counts before the initiation of pembrolizumab treatment may be useful in the identification of early-onset irAEs in clinical practice. Importantly, the early detection of irAEs could contribute to the minimization of the burden on patients' QOL. The mechanisms behind the presentation of irAEs have not been fully clarified. In addition, the mechanisms underlying the correlation of irAEs with the treatment outcomes associated with ICI use and the association between pretreatment peripheral blood biomarkers and early-onset irAEs remain unclarified 28, 29. PD-1 monoclonal antibodies interrupt the immune suppression. They activate CD8-positive T-lymphocytes in the tumor microenvironment. These activated CD8-positive T-lymphocytes attack not only the tumors but also cause irAEs, suggesting that these activated CD8-positive T-lymphocytes exert systemic actions 21. Taken together, we analyzed a potential rationale that an increase in lymphocyte counts and a change in related blood cell count ratios might help predict early-onset irAEs. The results of this study were the opposite of what we expected. Further basic *in vivo* and *in vitro* studies are warranted to clarify these mechanisms. In this report, we primarily focused on the association between routinely available blood cell count data and any irAEs using real-world data. These biomarkers, once validated, will generally be easily available and not require additional costs or setup for use in the clinical setting.

This study has several limitations. First, it was a retrospective, observational design. The patients' follow-up depended on clinicians' discretion. Additionally, the presence of information bias cannot be excluded. For the above reason, we performed multivariable analyses to reduce the effect of potential confounding factors associated with observational studies and with clinical differences in patients' characteristics. Nevertheless, unmeasured confounders could not be controlled during multivariable analyses, which are a major limitation of our study, since controlling these could affect the results. Patients with complications or prior history of chronic or recurrent autoimmune diseases and interstitial pulmonary diseases due to a safety administration of ICIs did not be included in the present study. The other underlying diseases and other treatments including steroids and immune-suppressants were unknown; and the use of these agents may affect the study results. However, there are no reports of relationship between irAEs and underlying diseases and other treatments including steroids and immune-suppressants until now; and the association between baseline use of corticosteroids and ICIs efficacy in a recent study is controversial 30. Second, we could not fully assess the grading of irAEs using the Common Terminology Criteria for Adverse Events version 4.0. Third, the sample size was small despite the multicenter design. Fourth, various types of irAEs, with the exception of those affecting the skin, were not commonly observed, thereby limiting our evaluation pertaining to which type of irAEs most strongly contribute to the correlation with pretreatment or posttreatment peripheral blood biomarkers. Thus, the findings of this study should be confirmed in a larger cohort study with an adequate sample size.

## Conclusion

This is the first multicenter study to demonstrate that pretreatment blood cell counts, such as ALC, NLR, LMR, and PLR are significantly associated with pembrolizumab-induced irAEs in patients with advanced NSCLC. The findings suggest that considering the peripheral blood cell count data before the initiation of pembrolizumab may be useful for identification of early-onset irAEs in clinical practice. We believe that the early detection and cautious management of irAEs onset can clinically help achieve maximum benefit in patients treated with pembrolizumab monotherapy within the 6-week study period. The findings of this study provide preliminary information on the association of pretreatment blood biomarkers with the early onset of any irAEs in Japanese patients with advanced NSCLC. These findings can likely be translated to other Asian populations, and highlight the need for additional research in this area.

## Supplementary Material

Supplementary figure S1.

## Figures and Tables

**Table 1:** Baseline patient characteristics

Patient characteristics	Total *n* = 92 (%)
Age, median [years, interquartile range]	60 (34-85)
Sex	
Male	64 (69.6)
Female	28 (30.4)
ECOG PS	
0	30 (32.6)
1	54 (58.7)
2	8 (8.7)
Treatment line	
1st	62 (67.4)
2nd	22 (23.9)
≥ 3rd	8 (8.7)
PD-L1 expression level	
Negative (< 1%)	1 (1.1)
Low (≥1%, < 50%)	17 (18.5)
High (≥50%)	74 (80.4)

Abbreviations: ECOG PS, Eastern Cooperative Oncology Group performance status; PD-L1, programmed cell death ligand 1.

**Table 2:** Immune-related adverse events during the first 6 weeks after the initiation of pembrolizumab treatment

Event	Total *n* = 92 (%)
Any irAEs	45 (48.9)
Skin reaction	35 (38.0)
Diarrhea	9 (9.8)
Thyroiditis/hypothyroidism	2 (2.2)
Adrenal insufficiency	2 (2.2)
Pneumonitis	1 (1.1)
Liver dysfunction	1 (1.1)
Hypophysitis	1 (1.1)
Neuropathy	1 (1.1)

Abbreviations: irAE, immune-related adverse event.

**Table 3:** Univariable and multivariable logistic regression analyses of the early onset of any immune-related adverse events

	Univariable analysis		Multivariable analysis							
Variables	Crude OR (95% CI)	*P*	Adjusted OR (95% CI)	*P*	Adjusted OR (95% CI)	*P*	Adjusted OR (95% CI)	*P*	Adjusted OR (95% CI)	*P*
Pretreatment ALC (> 1450)	0.34 (0.15-0.80)	0.014	0.24 (0.09-0.61)	0.003						
Pretreatment NLR (> 2.3)	5.81 (1.77-19.03)	0.004			5.99 (1.73-20.74)	0.005				
Pretreatment LMR (> 1.6)	0.14 (0.04-0.51)	0.003					0.12 (0.03-0.52)	0.004		
Pretreatment PLR (> 165)	3.05 (1.28-7.23)	0.011							2.87 (1.16-7.08)	0.022
Age	0.96 (0.93-1.00)	0.065	0.96 (0.92-1.00)	0.063	0.96 (0.92-1.00)	0.074	0.97 (0.93-1.01)	0.157	0.97 (0.93-1.01)	0.147
PD-L1 expression (≥50%)	4.35 (1.31-14.47)	0.017	5.79 (1.58-21.26)	0.008	3.59 (1.02-12.61)	0.046	4.86 (1.29-18.36)	0.020	3.98 (1.16-13.69)	0.028

Abbreviations: ALC, absolute lymphocyte count; NLR, neutrophil-to-lymphocyte ratio; LMR, lymphocyte-to-monocyte ratio; PLR, platelet-to-lymphocyte ratio; PD-L1, programmed cell death ligand 1; OR, odds ratio; CI, confidence interval. Multivariable logistic regression analysis adjusted for age and PD-L1 expression ( ≥ 50%) estimated the AUCs of pretreatment ALC, NLR, LMR, and PLR at 0.752, 0.718, 0.735, and 0.706, respectively.
