# Supplementary material for: Peripheral blood biomarkers predict immune-related adverse events in non-small cell lung cancer patients treated with pembrolizumab: a multicenter retrospective study
Source: J Cancer. 2021 Feb 16;12(7):2105–12. doi: 10.7150/jca.53242 (PMC7974524; doi:10.7150/jca.53242)
Supplement: Supplementary file 1 — Supplementary figure S1. [file jcav12p2105s1.pdf]

Pretreatment (A) ALC and (B) NLR

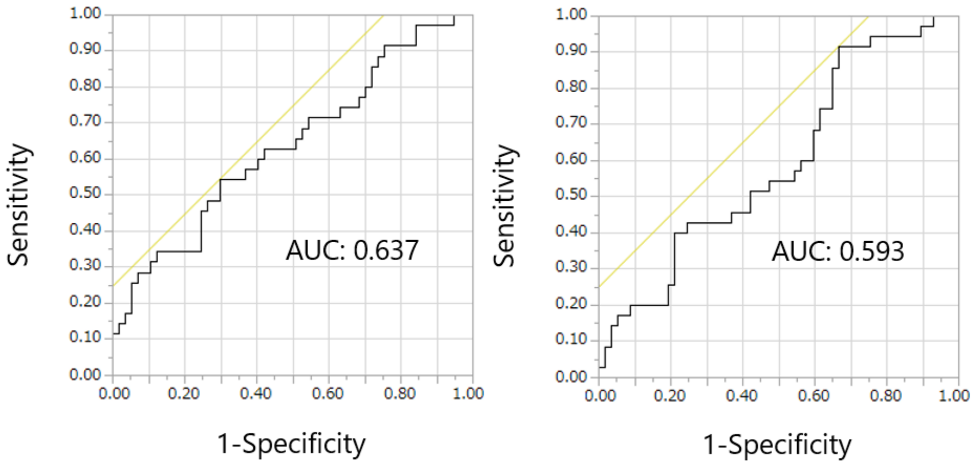

Pretreatment (C) LMR and (D) PLR

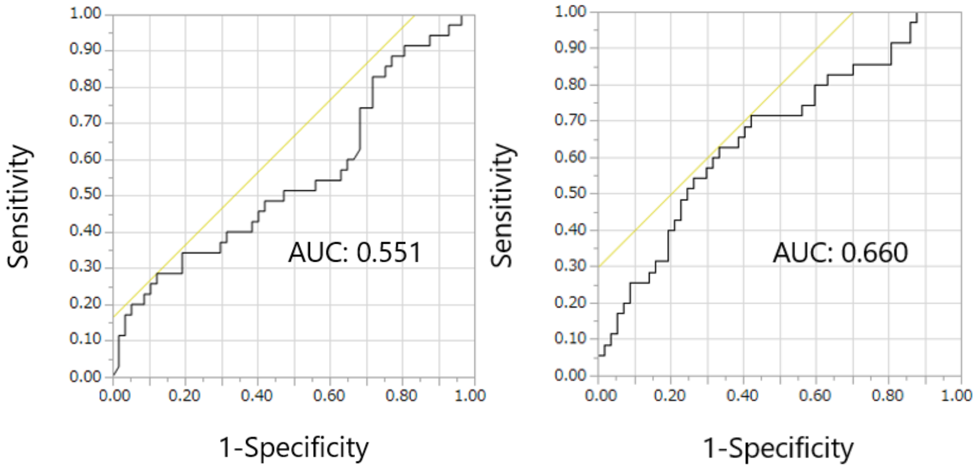

Post treatment (E) ALC and (F) NLR

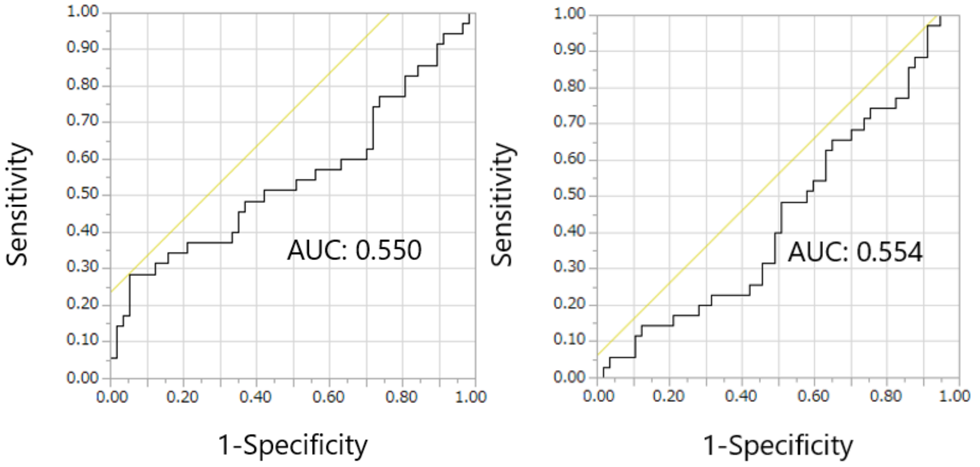

Post treatment (G) LMR and (H) PLR

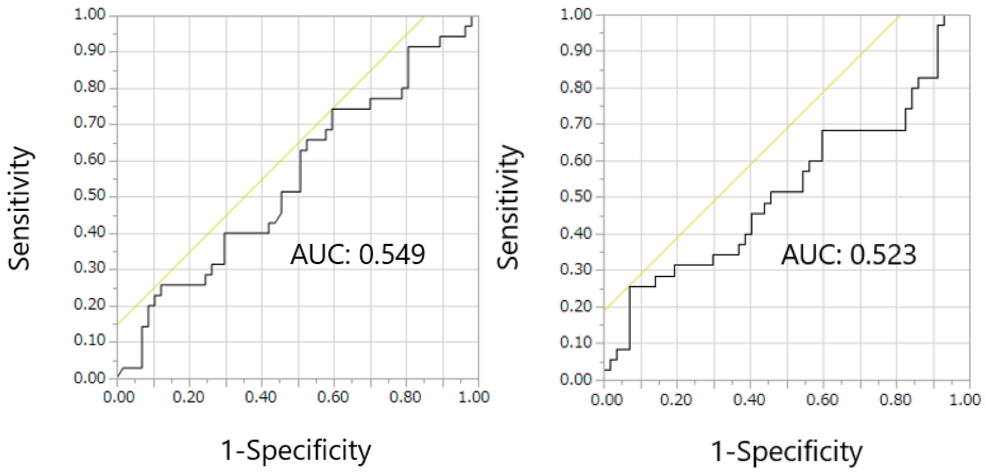

Post/pretreatment (I) ALC and (J) NLR

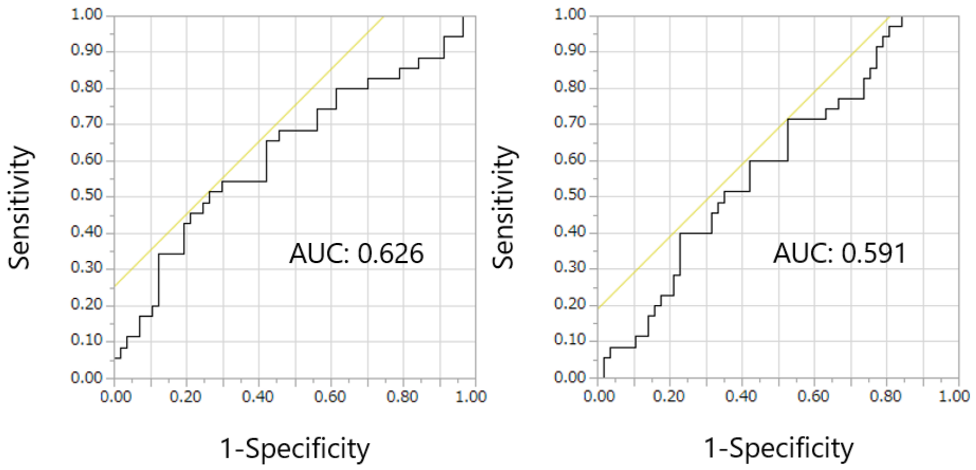

Post/pretreatment (K) LMR and (L) PLR

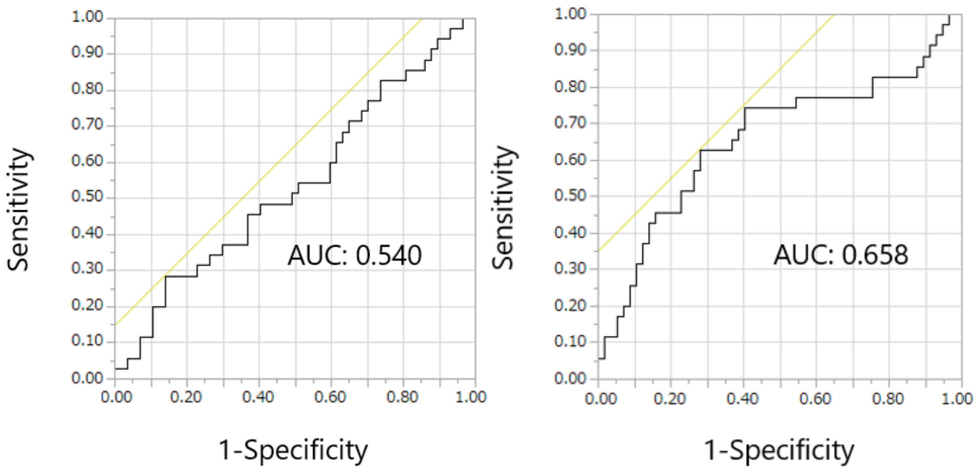

Supplemental online Figure 1
